# Intracardiac echocardiography–derived cavotricuspid isthmus morphology score for estimating procedural complexity in typical atrial flutter

**DOI:** 10.1093/europace/euag061

**Published:** 2026-04-15

**Authors:** Mustafa Gabarin, Majid Abonab, Juan Gabriel Acosta, Ziad Arow, Abdulrahman Alfraih, Guy Amit, Javier Bonacina, Mazin Alrasheed, Jason Roberts, Nigel Tan, Syamkumar Divakara Menon, Jorge A Wong, William F McIntyre

**Affiliations:** Division of Cardiology, Hamilton Health Sciences, Arrhythmia Service Unit, McMaster University, 237 Barton St. E, Hamilton, Ontario L8L 2X2, Canada; Cardiology Department, Meir Medical Center, Sackler Faculty of Medicine, Tel Aviv University, Kfar Saba, Israel; Division of Cardiology, Hamilton Health Sciences, Arrhythmia Service Unit, McMaster University, 237 Barton St. E, Hamilton, Ontario L8L 2X2, Canada; Division of Cardiology, Hamilton Health Sciences, Arrhythmia Service Unit, McMaster University, 237 Barton St. E, Hamilton, Ontario L8L 2X2, Canada; Cardiology Department, Meir Medical Center, Sackler Faculty of Medicine, Tel Aviv University, Kfar Saba, Israel; Division of Cardiology, Hamilton Health Sciences, Arrhythmia Service Unit, McMaster University, 237 Barton St. E, Hamilton, Ontario L8L 2X2, Canada; Division of Cardiology, Hamilton Health Sciences, Arrhythmia Service Unit, McMaster University, 237 Barton St. E, Hamilton, Ontario L8L 2X2, Canada; Division of Cardiology, Hamilton Health Sciences, Arrhythmia Service Unit, McMaster University, 237 Barton St. E, Hamilton, Ontario L8L 2X2, Canada; Division of Cardiology, Thunder Bay Regional Health Sciences Centre and NOSM University, Thunder Bay, Ontario, Canada; Division of Cardiology, Hamilton Health Sciences, Arrhythmia Service Unit, McMaster University, 237 Barton St. E, Hamilton, Ontario L8L 2X2, Canada; Division of Cardiology, Hamilton Health Sciences, Arrhythmia Service Unit, McMaster University, 237 Barton St. E, Hamilton, Ontario L8L 2X2, Canada; Division of Cardiology, Hamilton Health Sciences, Arrhythmia Service Unit, McMaster University, 237 Barton St. E, Hamilton, Ontario L8L 2X2, Canada; Division of Cardiology, Hamilton Health Sciences, Arrhythmia Service Unit, McMaster University, 237 Barton St. E, Hamilton, Ontario L8L 2X2, Canada; Division of Cardiology, Hamilton Health Sciences, Arrhythmia Service Unit, McMaster University, 237 Barton St. E, Hamilton, Ontario L8L 2X2, Canada; Division of Cardiology, Hamilton Health Sciences, Arrhythmia Service Unit, McMaster University, 237 Barton St. E, Hamilton, Ontario L8L 2X2, Canada

**Keywords:** Atrial flutter, Cavotricuspid isthmus, Intracardiac echocardiography, Radiofrequency ablation, Anatomical complexity score

## Abstract

**Graphical Abstract:**
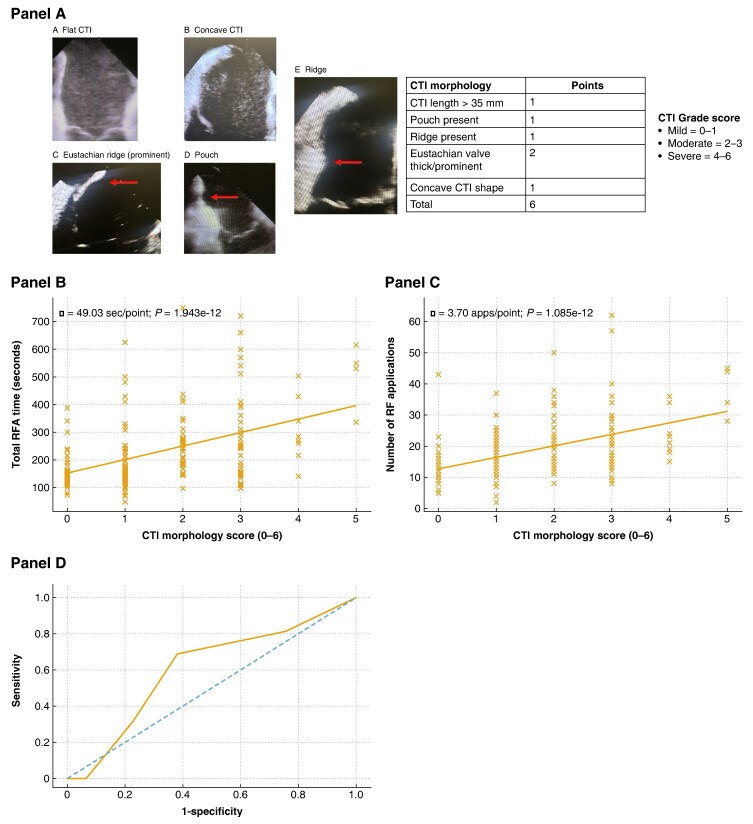
ICE-derived CTI morphology score and procedural outcomes. (*A*) Representative ICE images illustrating key CTI anatomical features used to derive the CTI morphology score, including flat vs. concave CTI, pouch formation, ridge, and prominent Eustachian ridge/valve. The scoring system (range 0–6) assigns points for CTI length > 35 mm (1), pouch (1), ridge (1), thick/prominent Eustachian ridge or valve (2), and concave CTI shape (1). Patients were categorized as mild (0–1), moderate (2–3), or severe (4–6) anatomical complexity. (*B*) Association between CTI morphology score and total RFA time. Each 1-point increase in CTI score was associated with a significant increase in ablation time (β ≈ 50 s per point; *P* < 0.001). (*C*) Association between CTI morphology score and number of radiofrequency applications delivered. Higher anatomical complexity correlated with increased lesion burden (≈3–4 additional applications per score point; *P* < 0.001). (*D*) Receiver operating characteristic curve evaluating the ability of the CTI morphology score to predict late AFL recurrence (>3 months), demonstrating modest discriminative performance (AUC ≈ 0.60). AFL, atrial flutter; CTI, cavotricuspid isthmus; ICE, intracardiac echocardiography; RFA, radiofrequency ablation; ROC, receiver operating characteristic.

ICE-derived CTI morphology score and procedural outcomes. (*A*) Representative ICE images illustrating key CTI anatomical features used to derive the CTI morphology score, including flat vs. concave CTI, pouch formation, ridge, and prominent Eustachian ridge/valve. The scoring system (range 0–6) assigns points for CTI length > 35 mm (1), pouch (1), ridge (1), thick/prominent Eustachian ridge or valve (2), and concave CTI shape (1). Patients were categorized as mild (0–1), moderate (2–3), or severe (4–6) anatomical complexity. (*B*) Association between CTI morphology score and total RFA time. Each 1-point increase in CTI score was associated with a significant increase in ablation time (β ≈ 50 s per point; *P* < 0.001). (*C*) Association between CTI morphology score and number of radiofrequency applications delivered. Higher anatomical complexity correlated with increased lesion burden (≈3–4 additional applications per score point; *P* < 0.001). (*D*) Receiver operating characteristic curve evaluating the ability of the CTI morphology score to predict late AFL recurrence (>3 months), demonstrating modest discriminative performance (AUC ≈ 0.60). AFL, atrial flutter; CTI, cavotricuspid isthmus; ICE, intracardiac echocardiography; RFA, radiofrequency ablation; ROC, receiver operating characteristic.

## Introduction

Catheter ablation of the cavotricuspid isthmus (CTI) is the first-line therapy for typical atrial flutter (AFL) and is associated with high acute success when bidirectional block is achieved.^1,2^ Despite these favourable outcomes, procedural workload varies substantially among patients. This variability is largely driven by heterogeneity in CTI anatomy, including differences in isthmus length, curvature, and the presence of anatomical obstacles such as pouches, ridges, and a prominent Eustachian ridge.^3,4^ These features can impair catheter contact and stability, necessitating additional ablation.^5^ The novelty of the present study lies in the standardization and semi-quantitative integration of established CTI anatomical features into a simple intracardiac echocardiography (ICE)–derived morphology score that translates real-time anatomy into expected procedural workload.

Intracardiac echocardiography, particularly when integrated with three-dimensional electroanatomical mapping, allows real-time visualization of CTI anatomy during ablation.^6,7^ While prior studies have linked CTI features to procedural difficulty, a simple quantitative framework to translate anatomical complexity into expected procedural workload is lacking.^8^ We therefore developed an ICE-derived CTI morphology score and evaluated its association with ablation workload and clinical outcomes during fluoroless CTI ablation.

## Methods

We conducted a retrospective observational study of 200 consecutive adult patients undergoing CTI ablation for typical AFL at a tertiary centre between January 2021 and April 2024. All procedures were performed using a fluoroscopy-free approach guided by ICE and a three-dimensional electroanatomical mapping system. Patients with aborted procedures or incomplete imaging or follow-up data were excluded.

Cavotricuspid isthmus anatomy was assessed using archived ICE recordings and three-dimensional reconstructions. Five predefined anatomical features were scored to generate a semi-quantitative CTI morphology score (range 0–6): CTI length > 35 mm (1 point), presence of ≥1 pouch (1 point), presence of a ridge (1 point), thick or prominent Eustachian ridge or valve (2 points), and concave CTI shape (1 point). Patients were categorized as having mild (0–1), moderate (2–3), or severe (4–6) anatomical complexity. Morphology assessment was performed independently by two experienced electrophysiologists blinded to procedural outcomes. Intracardiac echocardiography recordings and three-dimensional reconstructions were reviewed separately prior to comparison. No discrepancies were observed across the predefined scoring features, and therefore, formal statistical measures of interobserver agreement were not calculated.

Ablation was performed with an irrigated-tip catheter using high-power, short-duration lesions (50 W, 12 s), delivered in a linear fashion from the tricuspid annulus to the inferior vena cava. The procedural endpoint was bidirectional CTI block confirmed by differential pacing.

Primary procedural outcomes were total radiofrequency ablation (RFA) time and number of RF applications. Clinical outcomes included acute procedural success and late AFL recurrence (>3 months). Because a substantial proportion of CTI ablations were performed as part of combined procedures, most commonly in conjunction with pulmonary vein isolation, total procedure duration did not reliably reflect CTI-specific procedural complexity and was therefore not analysed. Associations between CTI score and outcomes were evaluated using linear and logistic regression, modelling the score per 1-point increase. Discrimination for late recurrence was assessed using receiver operating characteristic analysis.

## Results

The mean age of the cohort was 62.2 ± 10.1 years, and 70% were male. Cavotricuspid isthmus morphology grades were mild in 117 patients (58.5%), moderate in 71 (35.5%), and severe in 12 (6.0%). Acute bidirectional CTI block was achieved in 199 of 200 patients (99.5%).

Higher CTI morphology scores were strongly associated with increased procedural workload. On linear regression, each 1-point increase in score corresponded to an additional 53.4 s of RFA time (95% CI 42.7–64.1; *P* < 0.001) and 3.88 additional RF applications (95% CI 3.06–4.71; *P* < 0.001). The CTI score was not associated with acute procedural success (*P* = 0.19).

Late AFL recurrence occurred in 14 patients (7.0%). Each 1-point increase in CTI score was associated with a trend towards higher odds of late recurrence (OR 1.35, 95% CI 0.96–1.90; *P* = 0.085), with modest discriminative ability (AUC 0.60). The association between CTI morphology score and procedural workload remained consistent across standalone and combined procedures and did not differ between first-time and redo ablations. Complications were uncommon, with major bleeding in two patients (1.0%) and no strokes, tamponade, or procedure-related deaths.

## Discussion and conclusion

In this cohort of fluoroless, ICE-guided CTI ablations, a simple 0–6 CTI morphology score provided a reproducible, real-time measure of anatomical complexity that strongly predicted procedural workload. Each incremental anatomical feature was associated with approximately one additional minute of radiofrequency delivery and four extra lesions, while acute procedural success remained uniformly high. These findings underscore that CTI anatomy primarily influences procedural effort rather than bidirectional block and are consistent with prior anatomical and imaging studies linking isthmus length, recesses, and ridges to ablation difficulty rather than failure.^3–5,8^ Accordingly, the primary clinical purpose of the CTI morphology score is to anticipate procedural workload and technical complexity rather than to serve as a prognostic tool for long-term arrhythmic outcomes.

The modest discriminative performance of the score for late AFL recurrence, despite a trend towards higher odds of recurrence with increasing anatomical complexity, highlights the multifactorial nature of post-ablation outcomes. Durable lesion formation, gap recovery, catheter–tissue interaction, and the broader atrial substrate likely outweigh anatomical complexity alone in determining long-term rhythm control.^1,5,6^ This interpretation is supported by contemporary observational data demonstrating a high incidence of atrial fibrillation following typical flutter ablation, even when CTI block is durable.^9^ The FLUTFIB study further reinforces that late arrhythmia outcomes are largely driven by atrial disease progression rather than isthmus anatomy *per se*.^9^

From a procedural perspective, the CTI morphology score offers practical intra-procedural value. Early identification of complex anatomy may assist operators in anticipating ablation duration, lesion burden, and technical challenges, particularly in fluoroless workflows where ICE serves as the primary imaging modality.^7^ Rather than functioning as a prognostic tool for recurrence, the score translates real-time anatomical information into an expectation of procedural workload, thereby supporting planning, efficiency, and operator awareness.

This study has several limitations, including its single-centre design and the absence of external validation. Additionally, the CTI morphology score is based on predefined anatomical weighting rather than a data-derived model and therefore requires validation in independent cohorts.

In summary, an ICE-derived CTI morphology score provides a concise and reproducible framework for quantifying anatomical complexity during typical AFL ablation. While it does not independently predict late recurrence, it reliably anticipates procedural burden without compromising acute success. Integration of anatomical complexity with atrial substrate and lesion-quality metrics may further refine procedural planning and long-term risk assessment in future studies.

## Data Availability

The data underlying this article are available within the article. Additional data supporting the findings of this study are available from the corresponding author upon reasonable request. This study will be presented at the EHRA Congress 2026 (Paris, France) during the session ‘Atrial flutter ablation’ (Moderated ePosters), 14 April 2026, 13:30–14:15.
